# Infant Feeding

**Published:** 1888-12

**Authors:** 


### INFANT FEEDING.

Nature has provided the infant with a plentiful supply of sound and wholesome food, which in most cases answers its purpose admirably, and better than any substitute. Should the milk not suit the infant, the mother's health and habits should be inquired into, and an attempt should be made to restore the balance. Children under a year old should not be drugged, or at most they should be approached through the mother. After they are weaned their food should remain the simplest and least stimulating posssible. Instead of that what commonly takes place? The child of four months old is fed partly with pap or sop made of fermented and often sour bread, because, forsooth it is not satisfied with its natural food; then its health fails, and the mother gives it a teaspoonful of castor-oil; a little later the unfortunate infant is allowed to have meat, bacon, potatoes, biscuits, and cake, and it is no uncommon matter to see children of two or three years eating regularly quantities and kinds of food that would better suit little creatures of six or seven. Of course, the health suffers, and do what the mother can, her offspring gives her a world of trouble and anxiety, and she poor thing wonders how it can be so ill, as it is allowed to have everything it wants and in the largest amount. Up to the end of the first nine or ten months the mother ought to suffice; for the next six or eight, the diet should consist of milk freely diluted with a little milk-sugar. Still later, additions to the diet should be cautiously managed—fermented bread, plum-puddings, and meat being long withheld. I saw a little fellow a day or two ago ill for the second or third time lately from a surfeit of apples—raw, hard or indigestible; while a few weeks ago I was asked to pronounce an opinion on a little girl, four years old. Well, she was not very ill, her complaint was seven (or was it nine?) pieces of cake and jam, besides a few other light matters! Simple and sparing diet is what children ought to have, and nothing more, and it is the reverse of kindness to yield to the child's cries, and to permit it to gorge itself on food which it cannot assimilate.

